# "Step by Step". A feasibility study of a lunchtime walking intervention designed to increase walking, improve mental well-being and work performance in sedentary employees: Rationale and study design

**DOI:** 10.1186/1471-2458-10-578

**Published:** 2010-09-27

**Authors:** Cecilie Thøgersen-Ntoumani, Elizabeth A Loughren, Joan L Duda, Kenneth R Fox, Florence-Emilie Kinnafick

**Affiliations:** 1 School of Sport and Exercise Sciences, University of Birmingham, UK; 2 Department of Exercise, Nutrition and Health Sciences, University of Bristol, UK

## Abstract

**Background:**

Following an extensive recruitment campaign, a 16-week lunchtime intervention to increase walking was implemented with insufficiently physically active University employees to examine programme feasibility and the effects of the programme in increasing walking behaviour, and in improving well-being and work performance.

**Methods/design:**

A feasibility study in which participants were randomised to an immediate treatment or a delayed treatment control (to start at 10 weeks) group. For the first ten weeks of the intervention, participants took part in three facilitator-led group walks per week each of thirty minutes duration and were challenged to accumulate another sixty minutes of walking during the weekends. In the second phase of the intervention, the organised group walks ceased to be offered and participants were encouraged to self-organise their walks. Motivational principles were employed using contemporary motivational theory. Outcome measures (including self-reported walking, step counts, cardiovascular fitness, general and work-related well-being and work performance) were assessed at baseline, at the end of the 16-week intervention and (for some) four months after the end of the intervention. Process and outcome assessments were also taken throughout, and following, the intervention.

**Discussion:**

The results of the intervention will determine the feasibility of implementing a lunchtime walking programme to increase walking behaviour, well-being and performance in sedentary employees. If successful, there is scope to implement definitive trials across a range of worksites with the aim of improving both employee and organisational health.

**Trial registration:**

Current Controlled Trials ISRCTN81504663.

## Background

It is well established that regular physical activity is associated with a range of positive physical and psychological health benefits [1]. However, the majority of adults do not engage in sufficient levels of physical activity to sustain or improve health [2]. In recognition of the importance of physical activity to health and well-being in adults, the National Institute for Health and Clinical Excellence (NICE) recently published their guidance for supporting physical activity in the workplace in an effort to increase such behaviour among large segments of the adult population [3].

Sedentary individuals have the most to gain in terms of health from physical activity interventions, but tend to be more resistant to behaviour change. Accordingly, an increasing body of research has been focusing on the identification of effective strategies to increase physical activity among sedentary individuals, primarily through walking. Recent evidence indicates that it is possible to increase walking through interventions that are targeted and/or tailored to sedentary individuals that last twelve weeks [4]. Such programmes can increase the amount of walking by 30-60 minutes per week. This would contribute significantly to the national recommendations of 150 minutes of moderate intensity physical activity per week. Furthermore, a meta-analysis of pedometer-based walking interventions indicated modest weight loss (with programmes lasting an average of 16 weeks) and therefore prevention of further weight gain [5].

Although research is scarce, walking interventions have been successfully implemented in the work setting [6], and also among previously sedentary employees [7]. The workplace has been targeted as an important location for health promotion, particularly with regard to mental health [8] and also obesity prevention [9]. More direct benefits of physical activity to employers may accrue in terms of enhanced employee work satisfaction, performance and productivity. However, the evidence linking participation in employee exercise programmes with health, performance and well-being is inconclusive [10,11]. This is largely due to the health-conscious and active minority of employees (approximately 20%) tending to enrol in such programmes [12]. Thus, interventions are needed which centre on effectively recruiting to such programmes less physically active and more health needy employees.

The (relatively few) interventions that have tried to recruit such populations have shown some early signs of success. For example, Mutrie et al. [7] have shown that an intervention consisting of the provision of written materials, local information about walking routes and paths, and safety information, all targeted to the participants' stage of readiness for physical activity, can increase walking (as a form of active commuting to work) six months later among sedentary employees compared to a delayed treatment control group. However, lunchtime walking may be particularly suitable to employees for whom active commuting may be impractical (e.g., due to childcare responsibilities) or perceived to be too time-consuming.

Although work designed to identify effective ways of promoting physical activity among sedentary employees is very much still in its infancy, even less is known about the role of increasing lifestyle physical activity, such as walking, in well-being among employees. This is despite an expanding volume of work showing that physical activity is generally effective in improving mental well-being [13]. Previous research has demonstrated interrelationships between lifestyle physical activity, such as walking, work-related, and global dimensions of well-being [14]. Findings related to work-related well-being are pertinent in predicting both employee and organisational functioning, as such indicators (including positive affect at work and job satisfaction) are reliable predictors of work performance [15,16]. Previous studies in this area, however, have generally relied on cross-sectional designs and measured feeling states in a retrospective fashion, rather than in real-time. In the present study, we sought to measure states of work-related affect and satisfaction daily using an ecological momentary assessment (EMA) method made possible via the use of new technology (smartphones). We did this to examine fluctuations in job-related affect and satisfaction in the mornings and afternoons on days in which employees engaged in a lunchtime walking programme and on days when they did not.

In implementing motivational strategies, theory-based interventions can be very informative in terms of understanding *why *and *how *physical activity interventions work [17]. In brief, Self-Determination Theory (SDT) [18,19] suggests that people's reasons for engaging in an activity can be more or less autonomous (i.e., self-determined) or controlled. Such differences in motivation undergirding behavioural engagement have implications for motivation-related outcomes, including adherence. One the mini-theories of SDT, basic needs theory, suggests that satisfaction of basic human needs for autonomy, competence and relatedness will lead to more self-determined motivation, which in turn is associated with behavioural persistence, and higher levels of health and well-being. The theory specifies that environmental factors can be manipulated to facilitate satisfaction of the three needs. Indeed, recent intervention work has shown that it is possible to train exercise instructors to provide an autonomy-supportive exercise climate [20], and the ensuing satisfaction of the three needs can lead to more self-determined motivation, higher attendance and higher levels of positive mood in the exercisers [20]. This research needs to be expanded to examine whether the autonomy-supportive strategies delivered by exercise instructors can be equally effective when they are adapted to other exercise contexts such as the workplace.

Research has identified the particularly salient role of relatedness needs at the stage in which largely sedentary adults attempt to adopt physical activity [21]. This finding is complemented by research highlighting the importance of the provision of social support and promotion of group cohesion to levels of attendance in physical activity programmes [22]. For physical activity to be maintained in the long-term however, it is also necessary to develop a sense of autonomy, or self-determination, in participants. Thus, in this study, a group-based walking programme will be implemented in the first part of the intervention, accompanied by encouragement of additional home-based walking. We will examine the feasibility of withdrawing our organised group activities approximately half-way through the intervention to further encourage self-initiated walking or the formation of informal groups.

In summary, through this intervention, we posed the following research questions 1) which recruitment strategies are most effective in recruiting insufficiently physically active employees to a lunchtime walking programme?, 2) is it feasible to implement a 16-week lunchtime walking intervention with this population?, and 3) what are the effects of the intervention on walking behaviour, well-being and work performance?

## Methods and design

### Design

This randomised controlled trial sought to assess the feasibility and effectiveness of a sixteen-week lunchtime walking intervention to increase (and sustain) walking behaviour, improve general and work-related well-being, and enhance work performance levels in insufficiently physically active non-academic University employees. A sixteen-week randomised controlled design with a delayed treatment control group was used. The participants were randomised by means of a computer programme into an immediate treatment (*n *= 35) or a delayed treatment control (*n *= 40) condition. The latter group started the intervention programme in week 10 of the programme.

### Ethical approval

Ethical approval was obtained from the Life and Environmental Sciences Ethical Review Committee at the University of Birmingham. All participants were treated in accordance with principles put forward by the Helsinki declaration.

### Recruitment process

**Figure 1:**
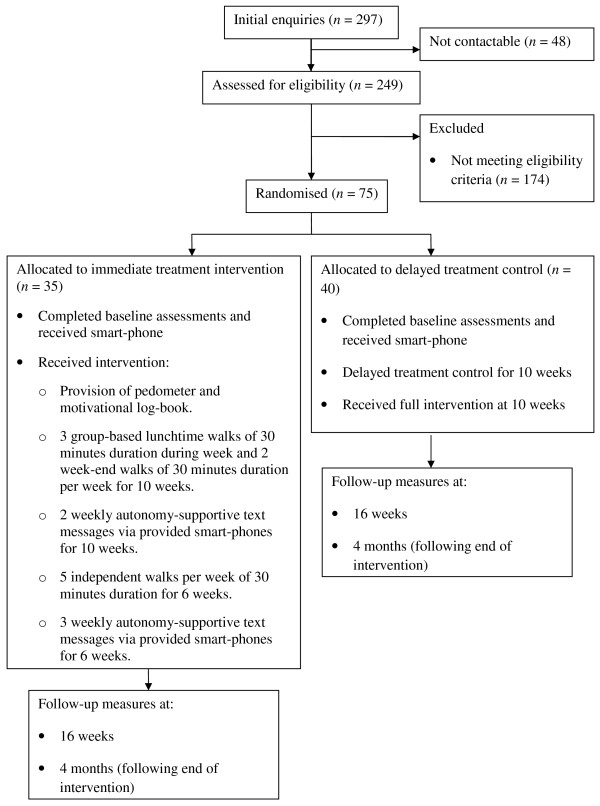
**Flowchart of participant recruitment and trial design**.

Using a range of strategies, recruitment took place between July and December 2009. Initially, interest in the programme was gauged through an open stall in a one-day health fair taking place at a large University in the West Midlands of the UK. Basic information about the intended programme was provided, and interested parties were encouraged to note down their e-mail details and were subsequently sent a link to an online survey (using the online software SurveyMonkey). Here, they were asked to provide further information on their physical activity levels using an expanded version of Godin and Shephard's Leisure-Time Exercise Questionnaire [23] (for information about the expanded version of the questionnaire, please contact the first author). If they were deemed eligible based on their physical activity levels (see "inclusion criteria" in the "Power calculation and study population" section), they were asked to also provide details about their date of birth, gender, job status, typical working hours, and completed a written informed consent. Additionally, an article in the staff University newspaper raising awareness of the programme was published and brief messages were provided on the back of all staff pay-slips and on electronic "totems" (information stands) located throughout the main University campus. Paragraphs about the programme were published in University-wide electronic newsletters and departmental newsletters. In addition, posters and flyers were strategically positioned in areas of the University where the research team and collaborators believed the target population would frequent (e.g., refectories, staff bar, main administrative centre of the University). We also provided information about the programme in University induction sessions for new staff and through a University web based information portal for all employees. Finally, a web-site targeted to interested participants was created and its web address was published through the various recruitment channels. The flow of participants through the recruitment and randomisation process is presented in Figure 1.

### Power calculation and study population

This was a feasibility trial as specified by the MRC guidelines for designing complex interventions. Consequently the sample size was determined by a consideration of the results of King, Ahn, Oliveira, Atienza, Castro, and Gardner [24] who reported a large effect of an 8-week physical activity intervention on minutes per week in moderate intensity physical activity. We also consulted the corporate partner to confirm a realistic target number for a feasibility study. Thus, we aimed to recruit a total sample of 68 participants given an effect size of *d *= .70, statistical power of 80% at a significance level of 5%, with a potential loss to follow-up of 25%.

Inclusion criteria for participation in the intervention were: healthy, mobile, 18-65 year old full-time employees who reported they were engaging in less than thirty minutes of moderate intensity physical activity on five days per week (i.e., insufficiently physically active). Exclusion criteria were employees with significant auditory or visual problems and those who had severe musculoskeletal disorders that prevented them from engaging in physical activity. Medical clearance was requested for those who reported any cardiovascular disease or back pain preventing them from exercising.

### The intervention

The intervention consisted of a group support phase (weeks 1-10) and an independence phase (weeks 11-16). Prior to the group support phase, nine qualified (i.e., already walk-leader trained by nationally recognised organisations, such as Natural England) walk leaders were recruited and trained via one two-hour workshop in the basic provision of an autonomy-supportive leadership style by a member of the research team.

In the workshop, the walk leaders were briefly introduced to the basic principles and tenets of SDT. The motivation-related relevance of why individuals engage in activities (such as physical activity) was discussed and the concepts of more or less autonomous in contrast to controlled reasons for behavioural engagement were described and illustrated. The walk leaders were then introduced to the construct of basic psychological needs as the fuel for more autonomous participation in physical activities as well as the well-being effects associated with an active lifestyle. The needs for competence, autonomy and relatedness were subsequently defined. Pulling in particular from previous intervention work centred on implementing an autonomy supportive exercise leader style [20], the discussion then turned to how walk leaders could promote participants' feeling more competent regarding walking behaviour, autonomous in their participation in the walking groups as well as self-directed walking, and more connected with other members of the walking group as well as with the walk leader. The walk leaders were encouraged to provide concrete examples of these principles based on their own or observed good practice.

During the group-support phase, the participants were asked to attend three weekly group lunchtime walks of thirty minutes duration, facilitated by one of the trained walk leaders (maximum 12 participants per group). Specifically, via the trial participant web-site, the participants were asked to sign up to three walks per week via a doodle registration site. The participants signed up to walks in advance for each two-week period for the first ten weeks (i.e., the group-support phase). A range of 30-minute walks (including special "themed" walks; all of which had been meticulously planned and tested by members of the research team) were offered for either 12.30 or 1.15 p.m. in and around the main University campus Mondays to Thursdays. The route for each of these walks had been mapped and could be viewed through the web-site.

The participants were also challenged to accumulate sixty minutes of walking during the week-ends. This would meet national recommendations of 150 mins/wk. The participants were provided with unsealed Yamax Digi-Walker 351 pedometers the week prior to the start of the intervention.

A motivational booklet was provided to the participants at the beginning of the intervention. Specifically, educational information about adoption and maintenance of physical activity (e.g., identifying/countering exercise barriers and goal setting principles) was provided in the booklet. Importantly, the motivational booklet also included an "Am I on track?" table, from which participants could interpret their weekly walking achievements through the provision of written feed-back (for the exact information provided, please see Table 1). Additionally, sections of this log-book requested the participants to indicate their reasons for walking, identification of their favourite walks, and the participants were asked which other places/areas they would like to walk. The participants were asked to retain this booklet for future use following the end of the intervention.

**Table 1:** Written feed-back on weekly attainment of walking achievements

Am I on track?		
**Total steps**	**Hours walked (per week)**	
>10,000-12,500	2.5	Excellent! Keep up the good work
>7,000-10,000	2.0	Well done! If you try to accumulate 30 more minutes per week, you will achieve the goals for health
5,000-7,000	1.0- < 2.0	You're off to a good start! Try to schedule regular walk times during your lunch hour and week-ends.
<5,000	<1.0	Don't give up! We understand it is difficult fitting in regular walks into your lifestyle. If you feel you need extra support, we are here to help. Please contact us.

The provision of autonomy-supportive text messages constituted another part of the intervention during both the group-based and the independent intervention phase. During the group-support phase, two weekly autonomy-supportive text messages (times were randomly allocated) were sent to the participants via a smart-phone (Nokia 2730 Classic) which was provided to the participants at the beginning of the intervention. SDT principles (e.g., offering choice, supporting individual volition, minimising pressure and control, acknowledging participants' perspectives and feelings, and providing a meaningful rationale for engaging in walking) informed the tone of the text messages.

During the 6-week independence phase, the walk groups ceased to be offered on a formal basis, but the participants were encouraged to form informal groups (if they so wished). Thus, throughout this period, the participants were not asked to sign up to any particular walks on the doodle registration site, but were encouraged to make use of the walk routes they had been made aware of during the group-support phase as well as explore new ones. In other words, the participants were requested during the independence phase to self-organise their walking routines with minimum help from the research team (however, the participants were still encouraged to contact the research team if they needed it). However, the participants were provided with three weekly autonomy-supportive text messages during this intervention period.

The delayed treatment control group started the full intervention after ten weeks. During the control period, the participants did not receive any of the intervention or intervention material as outlined above (e.g., pedometers, motivational booklets) and were not alerted to the trial web-site but were asked to continue their usual behaviours. They knew that they would be contacted in a few months regarding the start of their programme. They did receive the smart-phones at the beginning of their control period, as these phones were not only used as a motivational tool (i.e., by provision of weekly autonomy-supportive text messages during the intervention period) but also as a measurement device (see "outcome measures"). During the control period, weekly autonomy-supportive messages were *not *sent to the participants, but these only started once their intervention period began (as outlined for the immediate treatment group). Thus, during the control period for the delayed treatment control participants, the smart-phones were only used as a monitoring tool to assess work-related well-being.

### Outcome measures

The primary outcome measure was walking behaviour as assessed by the International Physical Activity Questionnaire [25]. This questionnaire was included as part of the baseline questionnaire package, and was administered again at 16 weeks and four months following the end of the intervention.

A related, but secondary, outcome measure was weekly step count as measured by the pedometer. The participants were not provided with the pedometers during a baseline period, as the researchers felt that the provision of the equipment would in itself work as an intervention and thus inflate walking behaviour. Thus, the weekly step counts were assessed only throughout the intervention period, which allowed comparisons across the different stages of the intervention (i.e., group phase and independent phase) to be made.

Adherence to the first ten weeks of the walking programme (except for week-ends) was assessed via registers taken by the walk leaders during the group phase of the intervention. In addition, a 16-week walking log was sent to the participants at the start of the intervention, in which they were asked to report daily the date of the walk, the number of 30-minutes walks per day, daily (and weekly) step count, and the walk route or area walked (for weeks 11-16, the log book also requested that participants noted the exact time of each 30-minute walk and if they walked alone or in groups, and if so, with whom). The walk log-book was returned to the research team at the end of the 16-week intervention.

Two kilometre field-based walking tests were conducted with each participant at baseline and at 16 weeks to assess changes in fitness status. Similar to the UKK walk test [26], the participants were instructed to walk 2 km (i.e. five laps) on an outdoor 400 metre track as fast as they could with a steady pace. After each lap, participants were provided prompts on the number of laps left to be completed. Time to complete the test was recorded by members of the research team.

General health and well-being, and work-related well-being scales constituted other secondary outcome measures. These included one item measuring current health perceptions (from the MOS SF-36; [27]), the Satisfaction With Life Scale [28], the Subjective Vitality scale [29], a job satisfaction scale [30], and the Job Affect Scale [31] which asks participants to rate their levels of affect during the past week and which can be categorised into four factors: enthusiasm, relaxation, nervousness and fatigue at work. Finally, the participants were asked to rate their own levels of work quality in the past four weeks using a 16-item instrument developed specifically for the present study, as well as one item measuring overall perceptions of work performance in the past four weeks taken from the WHO-HPQ [32]. The above scales were all administered via internal post at baseline, post-test (i.e., 16 weeks) and at the four-month follow-up.

Further, the participants were asked for their permission for members of the research team to contact their line managers to obtain pre and post measures of manager-rated work quality. The scale used to assess employee's work quality was similar to the self-report questionnaire constructed for the present study described above but consisted of eight items, as opposed to sixteen items. The item from the WHO-HPQ was also used to tap the managers' views about their respective employee's work quality in the previous four weeks. In addition, however, for more qualitative feedback, the managers were asked at baseline to describe the three most important characteristics of the targeted employee's job(s) and subsequently rate the employee on those characteristics. The manager questionnaire was administered at baseline and at 16 weeks.

An experience sampling approach was used to collect data pertaining to momentary work-related affect and job satisfaction states. The advantage of this approach is that it allowed us to measure well-being in real time, rather than relying on retrospective reports of feeling states. An added advantage of this method is that it is conducive to simultaneously examining relationships between variables both *between *and *within *participants. Once in the morning (between 10 and 11 a.m.) and once in the afternoon (between 2 and 3 p.m.) on two randomly chosen days (Monday-Thursday), the participants were prompted by a signal (and one reminder signal thirty minutes later if they had not responded on the first signal) on the provided smart-phone to complete the work-related state measures. The smart-phone devices were programmed to record the time of completion, and questions were presented in a random order to avoid order effects. The smart-phones were programmed to accept data once within a 30-minute window. The work-related well-being state scales inputted on the smart-phones included the Job Affect Scale [31] instructing participants to indicate how they felt at work at the moment of the signal and Judge and Ilies' [33] momentary job satisfaction scale. In addition, a single question relating to perceived daily work load was used. The scales were completed twice weekly for the duration of the intervention period (and including the control period for the delayed treatment control group).

Finally, individual differences measures that were used to control for in our analyses included the trait version of the Positive and Negative Affect Schedule [34], and the Work Extrinsic and Intrinsic Motivation Scale [35].

### Process measures

To examine the theoretically-assumed processes underpinning possible intervention effectiveness, process measures were obtained throughout the intervention period and at follow-up. Specifically, we assessed the participants' motivation for walking using the Behavioural Regulation in Exercise Questionnaire-2 [36], adapted to walking behaviour. This measure was administered via internal post in weeks 3, 10, 16 (i.e., end of the intervention period) and at the four month follow-up. We also assessed the participants' perceptions of the autonomy support provided by the walk leaders, text messages and the programme overall in weeks 3 and 10, using adaptations of the Health-Care Climate Questionnaire [37]. Finally, the participants' perceptions of psychological need satisfaction [38] derived from programme participation were measured in weeks 5, 10 and at the end of the intervention (i.e., week 16).

### Process evaluation

Apart from examining the effects of the intervention on outcomes, it was essential to conduct a process evaluation of the programme overall. We used both quantitative and qualitative methods to examine important feasibility aspects of the intervention.

Evaluations of the perceived effectiveness of the recruitment campaign, the walk routes and walk leaders were gathered via brief questionnaires at the end of week 10. At the end of week 16, the participants rated the acceptability and perceived effectiveness of the technological aspects of the intervention (i.e., smart-phones, text messages, pedometers, participant web-site, and the doodle registration site on which participants signed up to the walks). We also gathered quantitative information from those who dropped out from the programme, regarding their reasons for drop-out and acceptability of the intervention.

Focus group sessions and individual semi-structured interviews were conducted by a member of the research team (FK) with purposive selections of eight participants and all walk leaders at the end of the intervention period. The focus group interviews with the participants focused on the acceptability, reasons for adherence and barriers to participation in the intervention. The walk leader focus group interview centred on implementation and reflection of the SDT-based autonomy supportive principles, evaluation of the walk routes, resources created to be used with the programme (sign-in sheets, walk leader manual, on-line registration), and the perceived effectiveness of the walk programme as a whole.

To examine the extent to which the autonomy support training of the walk leaders worked, walk leaders wore microphones and carried audio-tape recorders on each walk during the ten-week period. Subsequently, five randomly chosen walk sessions for each walk leader were selected to code their provision of autonomy support. Two independent researchers subsequently rated those sessions and calculated an average of autonomy-support provision for each walk leader based on items used by Edmunds et al. [20].

### Analytical strategy

Research questions 1 and 2 will be addressed using descriptive statistical analyses to examine the effectiveness of the various components of the recruitment campaign, uptake of participants to the intervention, rates of adherence and drop-out. In addition, to further address research question 2, grounded theory [39] will be used to analyse the results of the semi-structured one-to-one and focus group interviews, and constant comparative analysis [40] will be adopted to interpret this data. Question 3 will be addressed using both ANOVA and Multilevel Modelling (MLM) analyses. Specifically, walking behaviour, well-being and work performance outcomes will be compared at baseline, post-test and 4-month follow-up between the intervention and control group using a repeated measures ANOVA design. Mixed design ANOVA analyses will be carried out for those measures which were assessed only twice. Multilevel Modelling (MLM) will be used to examine how within-person changes in the predictor variable (walking behaviour) predict within-person changes in the outcome variables (job affect and momentary job satisfaction). MLM has several advantages over repeated measures ANOVA. In particular, MLM can be used for the analysis of incomplete data [41]. The ANOVA analyses will be performed according to an intention-to-treat principle.

## Discussion

This article describes the rationale for and design of a feasibility study to increase walking, well-being and work performance in insufficiently physically active University employees. It used a randomised controlled design with a delayed treatment control group to examine the effect of the proposed lunchtime walking intervention on key outcomes. As one of the main limitations of workplace physical activity programmes has been the failure to attract the health needy proportions of the populations [12], we adopted a comprehensive recruitment campaign, working closely with the corporate partner, which allowed us to gain greater access to insufficiently physically active employees and thus those at risk of ill health. Walking programmes seem to be particularly well suited to less active populations [7] and by offering a lunchtime walking programme, employees who struggle to fit in sufficient levels of physical activity in their leisure-time (perhaps due to childcare commitments) may be more likely to adhere to a physically active lifestyle in the longer-term.

One of the other strengths of the intervention is that the outcomes did not only include self-report measures, but also manager reports as well as objectively assessed outcomes. Further, it employed an EMA approach in the measurement of work-related well-being to allow us to measure feeling states in real time, and to examine daily changes in job affect and satisfaction as a function of walking behaviour. To our knowledge, this is the first study to do that in this context using mobile technology.

In line with suggestions by other researchers [42], the design of the intervention was informed by established theory that may allow us to gain additional insight into reasons why the intervention works. Autonomy support through the programme was not only provided via (trained) walk leaders, but the smart phones also worked as a motivational tool to display autonomy-supportive text messages to the participants on a weekly basis. The latter approach has not previously been used, and thus extends research exploring innovative means of motivating less physically active employees to adopt and maintain a physically active lifestyle.

The intervention may benefit employees and employers alike. If the intervention proves successful, employee health and well-being can be enhanced. Further, our results will show whether the intervention appears to have an effect on manager-rated work performance and having healthier employees could also have positive implications for workplace absenteeism and presenteeism which will ultimately benefit the organisation as a whole. The results of the intervention (including the four-month follow-up) are expected in January 2011.

## List of abbreviations used

ANOVA: Analysis of Variance; EMA: Ecological Momentary Assessment; MLM: Multi Level Modelling; MRC: Medical Research Council; SDT: Self-Determination Theory; UKK: Urho Kaleka Kekkonen Walk Test; WHO-HPQ: World Health Organization-Health and Work Performance Questionnaire

## Competing interests

The authors declare that they have no competing interests.

## Authors' contributions

CTN, KRF, and JLD designed the study and wrote the initial protocol. JLD and FK delivered elements of the training programme. EL, with assistance from FK, co-ordinated the study with supervision from CTN and JLD. CTN drafted the manuscript. All authors read, provided comments and approved the final manuscript.

## Pre-publication history

The pre-publication history for this paper can be accessed here:

http://www.biomedcentral.com/1471-2458/10/578/prepub

## References

[B1] UK Department of Health At least five a week: Evidence on the impact of physical activity and its relationship to health: A report from the Chief Medical Officer 2004 London: Department of Health

[B2] UK Department of Health Health Survey for England, 2003 2004 London: National Statistics Office

[B3] National Institute for Health and Clinical Excellence Promoting physical activity in the workplace 2008 London: NICE

[B4] Ogilvie D Foster CE Rothnie H Cavill N Hamilton V Fitzsimons CF Mutrie N on behalf of the Scottish Physical Activity Research Collaboration (SPARColl) Interventions to promote walking: Systematic review BMJ 2007 334 1204 1213 10.1136/bmj.39198.722720.BE 17540909 PMC1889976

[B5] Richardson CR Newton TL Abraham JJ Sen A Jimbo M Swartz AM A Meta-Analysis of Pedometer-Based Walking Interventions and Weight Loss Ann Fam Med 2008 6 69 77 10.1370/afm.761 18195317 PMC2203404

[B6] Gilson N McKenna J Cooke C Experiences of route and task-based walking in a University community: Qualitative perspectives in a randomized control trial J Physical Activity Health 2008 5 Suppl. 1 S176 S182 10.1123/jpah.5.s1.s176 18364522

[B7] Mutrie N Carney C Blamey A Crawford F Aitchison T Whitelaw A "Walk in to work out": A randomised controlled trial of a self help intervention to promote active commuting J Epidemiol Community Health 2002 56 407 412 10.1136/jech.56.6.407 12011193 PMC1732165

[B8] Dame Carol Black's Review of the Health of Britain's Working Age Population Working for a Healthier Tomorrow 2008 London: TSO

[B9] UK Department of Health Healthy Weight, Healthy Lives A Cross Government Strategy for England 2008 London: Department of Health

[B10] Proper KI Koning M Van der Beek AJ Hildebrandt VH Bosscher RJ van Mechelen W The effectiveness of worksite physical activity programs on physical activity, physical fitness and health: Critical review Clin J Sport Med 2003 13 106 117 10.1097/00042752-200303000-00008 12629429

[B11] Proper KI Staal BJ Hildebrandt VH Van der Beek AJ Van Mechelen W Effectiveness of physical activity programs at worksites with respect to work-related outcomes Scand J Work Environ Health 2002 28 75 84 12019591 10.5271/sjweh.651

[B12] Grant CB Brisbin RE Workplace wellness: The key to higher productivity and lower health care costs 1992 New York: Van Nostrand Reinhold

[B13] Biddle SJH Fox KR Boutcher SH Physical activity and psychological well-being 2000 London: Routledge

[B14] Th∅gersen-Ntoumani Fox KR Ntoumanis N Relationships between exercise and three components of mental well-being in corporate employees Psychol Sport Exerc 2005 6 609 627 10.1016/j.psychsport.2004.12.004

[B15] Judge TA Thoresen CJ Bono JE Patton GK The job satisfaction-job performance relationship: A qualitative and quantitative review Psychol Bull 2001 127 376 407 10.1037/0033-2909.127.3.376 11393302

[B16] Rust R Discriminant validity of the "big five" personality traits in employment settings Soc Behav Pers 1999 27 99 108 10.2224/sbp.1999.27.1.99

[B17] Baranowski T Anderson C Carmack C Mediating variable framework in physical activity interventions: How are we doing? How might we do better? Am J Prev Med 1998 15 266 297 10.1016/S0749-3797(98)00080-4 9838973

[B18] Deci EL Ryan RM Intrinsic motivation and self-determination in human behavior 1985 New York: Plenum

[B19] Ryan RM Deci EL Self-Determination Theory and the facilitation of intrinsic motivation, social development, and well-being Am Psychol 2000 55 68 78 10.1037/0003-066X.55.1.68 11392867

[B20] Edmunds J Ntoumanis N Duda JL Testing a self-determination theory-based teaching style intervention in the exercise domain Eur J Soc Psychol 2008 38 375 388 10.1002/ejsp.463

[B21] Edmunds J Ntoumanis N Duda JL Adherence and well-being in overweight and obese patients referred to an exercise on prescription scheme: A Self-Determination Theory perspective Psychol Sport Exerc 2007 8 722 740 10.1016/j.psychsport.2006.07.006

[B22] Fraser SN Spink KS Examining the role of social support and group cohesion in exercise compliance J Behav Med 2002 25 233 249 10.1023/A:1015328627304 12055775

[B23] Godin G Shephard RJ A simple method to assess exercise behavior in the community Can J Appl Sport Sci 1985 10 141 146 4053261

[B24] King AC Ahn DK Oliveira BM Atienza AA Castro CM Gardner CD Promoting physical activity through hand-held computer technology Am J Prev Med 2008 34 138 142 10.1016/j.amepre.2007.09.025 18201644 PMC2715220

[B25] Craig CL Marshall AL Sjostrom M Bauman AE Booth ML Ainsworth BE Pratt M Ekelund U Yngve A Sallis JF Pekka O International physical activity questionnaire: 12-country reliability and validity Med Sci Sports Exerc 2003 35 1381 1395 10.1249/01.MSS.0000078924.61453.FB 12900694

[B26] Laukkanen RMT Kukkonen-Harjula TK Oja P Pasanen ME Vuori IM Prediction of Change in Maximal Aerobic Power by the 2-km Walk Test after Walking Training in Middle-Aged Adults Int J Sports Med 2000 21 113 116 10.1055/s-2000-8872 10727071

[B27] Ware JE Sherbourne CD The MOS 36-item short-form health survey (SF-36) Med Care 1992 30 473 483 10.1097/00005650-199206000-00002 1593914

[B28] Diener E Emmons RA Larsen RJ Griffin S The satisfaction with life scale J Pers Assess 1985 49 71 75 10.1207/s15327752jpa4901_13 16367493

[B29] Bostic TJ Rubio DM Hood M A validation of the Subjective Vitality Scale using structural equation modeling Soc Indic Res 2000 52 313 324 10.1023/A:1007136110218

[B30] Judge TA Locke EA Durham CC Kluger AK Dispositional effects on job and life satisfaction: The role of core evaluations J Appl Psychol 1998 83 17 34 10.1037/0021-9010.83.1.17 9494439

[B31] Brief AP Burke MJ George JM Robinson BS Webster J Should negative affectivity remain an unmeasured variable in the study of job stress? J Appl Psychol 1988 73 193 198 10.1037/0021-9010.73.2.193 3384771

[B32] Kessler RC Barber C Beck A Berglund P Cleary PD McKenas D Pronk N Simon G Stang P Ustun TB Wang P The World Health Organization Health and Work Performance Questionnaire (HPQ) J Occup Environ Med 2003 45 156 174 10.1097/01.jom.0000052967.43131.51 12625231

[B33] Judge TA Ilies R Affect and job satisfaction: A study of their relationship at work and at home J Appl Psychol 2004 89 661 673 10.1037/0021-9010.89.4.661 15327352

[B34] Watson D Clark LA Tellegen A Development and validation of brief measures of positive and negative affect: The PANAS scales J Pers Soc Psychol 1988 54 1063 1070 10.1037/0022-3514.54.6.1063 3397865

[B35] Tremblay MA Blanchard CM Taylor S Pelletier LG Work Extrinsic and Intrinsic Motivation Scale: Its value for organizational psychology research Can J Behav Sci 2009 41 213 226

[B36] Markland D Tobin V A modification to the behavioural regulation in exercise questionnaire to include an assessment of amotivation J Sport Exerc Psychol 2004 26 191 196

[B37] Williams GC Grow VM Freedman Z Ryan RM Deci EL Motivational predictors of weight loss and weight-loss maintenance J Pers Soc Psychol 1996 70 115 126 10.1037/0022-3514.70.1.115 8558405

[B38] Baard PB Deci EL Ryan RM Intrinsic need satisfaction: A motivational basis of performance and well-being in two work settings J Appl Soc Psychol 2004 34 2045 2068 10.1111/j.1559-1816.2004.tb02690.x

[B39] Strauss A Corbin J Basics of qualitative research, grounded theory procedures 1990 London: Sage

[B40] Janesick VJ Denzin NK, Lincoln YS The dance of qualitative research design: Metaphor, methodolatry, and meaning Handbook of qualitative research 1998 Thousand Oaks, CA: Sage 209 219

[B41] Singer JD Willet JB Applied longitudinal data analysis: Modeling change and event occurrence 2003 Oxford: University Press

[B42] Baranowski T Anderson C Carmack C Mediating variable framework in physical activity interventions: How are we doing? How might we do better? Am J Prev Med 1998 15 266 297 10.1016/S0749-3797(98)00080-4 9838973

